# Characteristics of interventions aimed at reducing inequalities along the cancer continuum: A scoping review

**DOI:** 10.1002/ijc.35478

**Published:** 2025-05-26

**Authors:** Wende C. Safari, Katja Gravenhorst, Clemence Leyrat, Koki Shimizu, Matthew J. Smith, Ajay Aggarwal, Camille Maringe

**Affiliations:** ^1^ Inequalities in Cancer Outcomes Network, Department of Health Services Research and Policy Faculty of Public Health and Policy, London School of Hygiene & Tropical Medicine UK; ^2^ Department of Medical Statistics Faculty of Epidemiology and Population Health, London School of Hygiene & Tropical Medicine UK; ^3^ Department of Health Services Research and Policy Faculty of Public Health and Policy, London School of Hygiene & Tropical Medicine UK; ^4^ Department of Oncology Guy's and St Thomas' NHS Foundation Trust London UK

**Keywords:** cancer continuum, cancer inequalities, experimental designs, interventions

## Abstract

Cancer inequalities are wide and enduring, within countries between socio‐demographic groups and between countries. These are generated and sustained throughout the key phases of the cancer pathway, from investigation, clinical assessment, decision and access to treatment, and follow‐up care. We aimed to describe the characteristics of implemented interventions, evaluated in published controlled experiments in the medical literature, specifically designed to target reductions in inequalities along the cancer pathway. We searched the Ovid Medline and Embase databases from January 2005 to April 2024 for controlled experiments reporting on interventions tackling inequalities. We extracted information on the publication, the aim and type of intervention, its setting, the characteristics of the sample and of the interventions, and summarised their results and limitations. We identified 56 articles reporting on 57 interventions. Of these, 51 (89.5%) focused on access to screening; 56 (98.2%) focused on colorectal, breast, and cervical cancers; 37 (64.9%) concentrated on ethnic inequalities and 48 (84.2%) were based in the USA. In addition, the majority of interventions sought to change individual knowledge, beliefs, and behaviour rather than issues at the system‐level. The importance of addressing how healthcare is delivered equitably to all individuals is widely recognised, and there is evidence that individual factors account for only a small part of cancer pathway inequalities. Yet, this scoping review reports a lack of diversity in the implementation of interventions addressing cancer inequalities, and a minority of them target health system issues.

## Graphical abstract

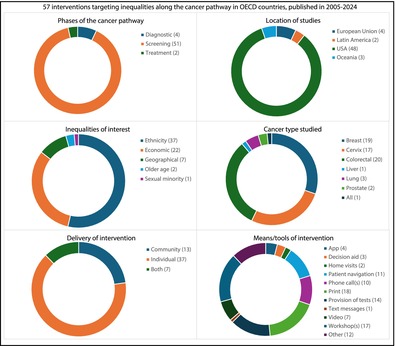

**Abbreviations**
CONSORT: Consolidated Standards of Reporting Trials
HUHY: ‘Help Us Help You’
ICON: Inequalities in Cancer Outcomes Network
IQR: interquartile range
MESH: Medical Subject Headings
OECD: Organisation for Economic Co‐operation and Development
PPIE: Public and Patient Involvement and Engagement
UK: United Kingdom
USA: United States of America

## 1 INTRODUCTION

The diversity and complexity of cancer pathways (from investigation and diagnosis to treatment and beyond) and the amount of resources involved often correlate with inequalities in access and use of cancer care^1^, ^2^, ^3^ and in outcomes,^4^ both by socio‐demographic characteristics within countries,^5^, ^6^, ^7^ and between countries.^8^ These inequalities manifest and accumulate throughout the different key phases of the cancer pathway from cancer investigation,^3^, ^9^, ^10^, ^11^, ^12^ to clinical assessment,^13^ decision to treat, access to treatment^1^ and follow‐up care.^14^, ^15^ In contrast to standard pathways designed at the national level to streamline patients according to symptoms, stage of disease, or treatment options, patients will have different experiences due to complex interplays between their socio‐demographic characteristics, their places of residence, diagnosis, and treatment, and how healthcare is delivered, such as allocation and management of resources.^13^, ^16^ These varying experiences often reflect inequity, whereby patients who have greater needs are not provided with additional support, or could hint at active opportunities for targeted interventions where inequalities are documented. While there is a wide range of health interventions targeting cancer awareness (e.g., the ‘Be Clear on Cancer’ and ‘Help Us Help You’ [HUHY] campaigns in England),^17^, ^18^ screening uptake, faster referrals, streamlining access to treatment, and enhanced post‐operative care, they may inadvertently create, amplify, or perpetuate inequalities if they are not designed carefully,^19^ for instance, if they do not embed specific elements that would recognise and address inequalities upfront. These inequalities amount to avoidable gaps in cancer outcomes as well as worsening mental health outcomes and poorer quality of life.

The aim of the present scoping review is to describe the characteristics of interventions, evaluated by controlled experiments and published in the medical literature, that are specifically designed to reduce inequalities along the cancer continuum.

### 1.1 Rationale

The Inequalities in Cancer Outcomes Network (ICON) is a group of researchers, based at the London School of Hygiene & Tropical Medicine (UK), that aims to reduce inequalities in access to cancer care, ensuring that everyone, regardless of their socio‐demographic background, can access care and benefit from improvements in cancer care.^20^ To apprehend the diversity of interventions in cancer equity research and benefit from its learnings to date, we scoped the literature for interventions that specifically aimed at eliminating or reducing inequalities in access to cancer care. Specifically, we aimed to understand the types of inequalities tackled, the types of interventions, the stakeholders involved, the target populations of these interventions, the settings of delivery, and how patients and the public contributed to their development.

That knowledge will help identify gaps in the literature as well as maximise our learnings to develop relevant interventions and strategies to address existing inequalities. We are aware that much literature on health inequalities in general, and cancer inequalities in particular, points to where these inequalities occur, their size, and often fails to offer solutions to address them. This scoping review gathers evidence from controlled experiments of interventions aiming to address these inequalities so as to inform the design of future tailored interventions.

## 2 DATA SOURCES AND SEARCHES

### 2.1 Article selection

We considered the literature that pertained to the experimental study of targeted interventions on inequalities along the cancer pathway, restricted to solid tumours in adults, in countries part of the Organisation for Economic Co‐operation and Development (OECD). Specific sets of key words for the selection of articles pertaining to inequalities, interventions, solid tumours, randomised controlled trial design, and OECD countries were defined and are provided in Appendix S1.

We defined *inequalities* as the unequal access to cancer services along the cancer continuum, in relation to someone's socio‐demographic characteristics such as socioeconomic background, ethnicity, gender, age, comorbidities, rurality, etc. We considered cancer investigation such as screening, diagnosis—through primary and secondary care, treatment, post‐treatment follow‐up, and palliative care as phases along the cancer journey, at which inequalities may arise or compound.

By *interventions*, we referred to any changes in service delivery along the cancer journey that involved a practical change in, for example, behaviour, delivery, or offer of a service. For this scoping review, we were not interested in the cancer treatments themselves.

Our focus was on articles that described and formally evaluated specific interventions through controlled experiments. Non‐solid tumours such as lymphoma and leukaemia were excluded as their investigation, diagnosis, and treatment are different from those of solid tumours. We searched Ovid Medline and Embase using the search terms provided in Appendix S1, with searches executed in April 2024. These two databases were selected for their complementarity: Embase includes medRxiv, while Ovid Medline is easier to search than PubMed, given the absence of a specific MESH term for our review. To refine the scope, we limited our review to articles published in English since January 2005. This 20‐year window helps capture a wealth of interventions as well as ensure contemporaneity for a transfer of the learnings to the present clinical practice and to OECD member countries, a proxy for the identification of high income countries.

In addition, the following exclusion criteria were used to assess the relevance of each article to the aim of the scoping review:

1. Type of article: (i) Conference abstract, (ii) Book review, (iii) Protocols, (iv) Expert reviews, and (v) Literature reviews;
2. Inadequate focus: (i) Drug trials, or trials of medical procedures or therapies, (ii) Tackling inequality in recruitment of trial participants, (iii) Not focused on cancer, (iv) Not focused on solid malignancies, (v) Children's cancer, and (vi) No intervention;
3. Inequalities outside the scope of our research, such as studies where: (i) No obvious inequality to resolve, (ii) Addressing international inequalities, (iii) Inequalities in prevention of cancer, (iv) Inequalities that are not on the direct cancer pathways such as financial hardship, mental health, and infertility, and (iv) Interventions that do not result in practical change in cancer service delivery.

Six of the co‐authors were involved in screening articles at all stages of article selection: title, abstract, and full text. Abstract and full text reviews were performed in pairs by co‐authors, blinded to each other's decisions, and when necessary, reconciliation happened through a third independent co‐author. The review and selection process were done using the online software Rayyan. We provide both the protocol for our scoping review and the PRISMA‐ScR checklist in Appendices S2 and S3.

### 2.2 Data extraction

We extracted information related to the article itself (authors, title, year of publication, and journal), the aim of the study and the type of study design, the setting (geographical location, recruitment period, length of follow‐up, setting of delivery, and sample size), the characteristics of the sample (cancer sites, socio‐demographic characteristics of individuals), the characteristics of the interventions (type of inequality, cancer phase, level of intervention, delivery type, description of the intervention includingmeans used and outcomes measured) and summaries of results and discussions (Appendix S4). We stored the information in Excel before transferring it to Stata for description and analysis.

We contrasted two types of delivery of interventions: individual‐based, where the intervention was directed at unique individuals; and community‐based, where the intervention was targeted at groups or communities. We also considered whether the interventions led to additional steps/burden on individuals (e.g., leaflets, education, and home‐tests), or whether interventions aimed to alleviate that burden (e.g., navigation, low or no cost services, and mobile clinics/units).

We tested the extraction checklist on five randomly selected articles and made amendments and improvements to the information captured. To ensure consistency in data extraction between authors, all initially extracted information from these five articles was reviewed. Agreement in the resulting extraction was high. Data extraction for the other articles was completed by a minimum of two people, who independently extracted information before checking and reconciling in pairs. Discrepancies were resolved by a third independent co‐author. The information extracted provides insights into the characteristics of interventions addressing inequalities, summarises their benefits and highlights gaps in the literature.

No ethical approval was necessary for the conduct of this research as only published aggregated data were used.

## 3 RESULTS

### 3.1 Search and selection of articles

**FIGURE 1:**
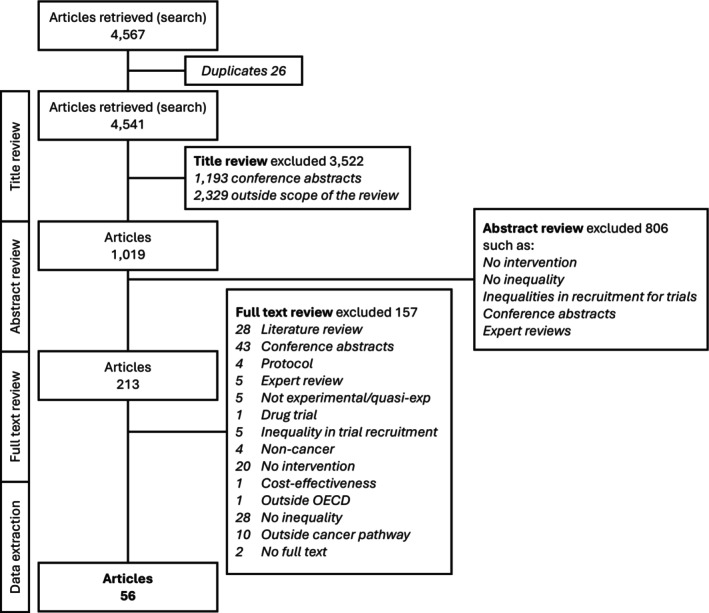
Flow chart for the selection and review of manuscripts relevant to the review.

We retrieved 4567 articles. Twenty‐six articles were duplicates and were excluded. Title review excluded 3522 articles, 1193 of them were conference abstracts. A further 806 articles were excluded after abstract review. Of the 213 remaining articles, we excluded 157 articles, retaining 56 for analysis reporting on 57 different interventions (Figure 1 and Table S1). One article reported on two different interventions.^21^

### 3.2 Characteristics of the studies

The main characteristics of the studies, mostly published post‐2010, are provided in Table 1, by phase of the cancer pathway as well as overall.

**TABLE 1:** General description of the 57 interventions studied by cancer phase.

Phase	Diagnostic		Screening		Treatment		All phases	
	*N*	%	*N*	%	*N*	%	*N*	%
*N* (row %)	4	7.0	51	89.5	2	3.5	57	100.0
**Year of publication**								
2005–2009	0	0.0	7	13.7	0	0.0	7	12.3
2010–2014	2	50.0	17	33.3	1	50.0	20	35.1
2015–2019	0	0.0	16	31.4	0	0.0	16	28.1
2020–2024	2	50.0	11	21.6	1	50.0	14	24.6
**Continent (place of study)**								
European Union	1	25.0	2	3.9	1	50.0	4	7.0
South and Central America	0	0.0	2	3.9	0	0.0	2	3.5
United States of America	3	75.0	44	86.3	1	50.0	48	84.2
Oceania	0	0.0	3	5.9	0	0.0	3	5.3
**Cancers of interest**								
Breast	3	75.0	15	29.4	1	50.0	19	33.3
Cervix	0	0.0	17	33.3	0	0.0	17	29.8
Colorectal	1	25.0	18	35.3	1	50.0	20	35.1
Liver	0	0.0	1	2.0	0	0.0	1	1.8
Lung	0	0.0	3	5.9	0	0.0	3	5.3
Prostate	0	0.0	2	3.9	0	0.0	2	3.5
All	1	25.0	0	0.0	0	0.0	1	1.8
**Inequality of interest**								
Ethnicity	3	75.0	33	64.7	1	50.0	37	64.9
Economic	2	50.0	20	39.2	0	0.0	22	38.6
Geographical	0	0.0	7	13.7	0	0.0	7	12.3
Older age	0	0.0	1	2.0	1	50.0	2	3.5
Sexual minority	0	0.0	1	2.0	0	0.0	1	1.8
**Comparison group**								
Active comparator	0	0.0	23	45.1	0	0.0	23	40.4
Usual care	3	75.0	25	49.0	2	100.0	30	52.6
No control	1	25.0	3	5.9	0	0.0	4	7.0
**Recruitment period (months, mean, SD)**	28	16.4	22.5	19.6	45.5	12.0	24.1	19.5
**Follow‐up period (months, mean, SD)**	5	1.7	8.5	8.4	9.0	4.2	8.3	8.1
**Cluster randomised trials**								
Nb of treatment clusters (mean, SD)	6	1	16	13	42	0	16	14
Nb of control clusters (mean, SD)	5	0	15	11	67	0	17	17
Sample size treatment group (mean, SD)	579	120	510	612	251	0	502	553
Sample size control group (mean, SD)	625	112	541	593	229	0	532	538
**Randomised trials (no clusters)**								
Sample size treatment group (mean, SD)	138	29	1585	4208	77	0	1478	4066
Sample size control group (mean, SD)	100	25	1180	2894	76	0	1094	2790
**Contribution of the study population (Y)**	4	100.0	33	64.7	0	0.0	37	64.9
**PPIE (Y)**	4	100.0	31	60.8	1	50.0	36	63.2
**Primary outcome benefit**								
Y	1	25.0	36	70.6	2	100.0	39	68.4
N	3	75.0	13	25.5	0	0.0	16	28.1
NA	0	0.0	2	3.9	0	0.0	2	3.5
**Secondary outcome benefit**								
Y	1	25.0	16	31.4	1	50.0	18	31.6
N	1	25.0	12	23.5	0	0.0	13	22.8
NA	2	50.0	23	45.1	1	50.0	26	45.6

Most interventions were targeting the screening phase (51 of 57, 89.5%),^21^, ^22^, ^23^, ^24^, ^25^, ^26^, ^27^, ^28^, ^29^, ^30^, ^31^, ^32^, ^33^, ^34^, ^35^, ^36^, ^37^, ^38^, ^39^, ^40^, ^41^, ^42^, ^43^, ^44^, ^45^, ^46^, ^47^, ^48^, ^49^, ^50^, ^51^, ^52^, ^53^, ^54^, ^55^, ^56^, ^57^, ^58^, ^59^, ^60^, ^61^, ^62^, ^63^, ^64^, ^65^, ^66^, ^67^, ^68^, ^69^, ^70^ with only 4 (7.0%) targeting the diagnostic phase^71^, ^72^, ^73^, ^74^ and 2 (3.5%) the treatment phase.^75^, ^76^ Three cancer sites were the focus of 98.2% of interventions; these were colorectal (20 interventions, 35.1%),^22^, ^23^, ^33^, ^37^, ^39^, ^40^, ^41^, ^43^, ^44^, ^46^, ^47^, ^49^, ^51^, ^55^, ^63^, ^64^, ^68^, ^70^, ^74^, ^76^ breast (19 interventions, 33.3%)^29^, ^30^, ^31^, ^35^, ^38^, ^42^, ^43^, ^48^, ^50^, ^52^, ^54^, ^57^, ^61^, ^62^, ^66^, ^71^, ^73^, ^74^, ^75^ and cervical (17 interventions, 29.8%)^24^, ^25^, ^26^, ^27^, ^28^, ^29^, ^32^, ^34^, ^42^, ^45^, ^53^, ^56^, ^58^, ^60^, ^65^, ^67^, ^69^ cancers, all of which have reliable tests for early detection and well established screening programmes. Other cancers of focus included lung,^21^, ^43^ prostate,^43^, ^59^ and liver^36^ with 3, 2, and 1 interventions respectively, all of which addressed inequalities in access to screening (Table 1).

The large majority of interventions were implemented in the USA (48 out of 57, 84.2%)^21^, ^22^, ^23^, ^25^, ^26^, ^27^, ^28^, ^29^, ^31^, ^33^, ^34^, ^35^, ^36^, ^37^, ^39^, ^40^, ^41^, ^42^, ^43^, ^44^, ^45^, ^46^, ^47^, ^48^, ^49^, ^51^, ^52^, ^54^, ^55^, ^56^, ^57^, ^59^, ^60^, ^61^, ^62^, ^63^, ^64^, ^65^, ^66^, ^67^, ^68^, ^69^, ^70^, ^71^, ^73^, ^74^, ^75^ and mostly addressed inequalities by ethnicity/race (37 interventions, 64.9%).^22^, ^23^, ^24^, ^25^, ^26^, ^27^, ^28^, ^29^, ^31^, ^33^, ^34^, ^36^, ^37^, ^41^, ^42^, ^43^, ^48^, ^50^, ^51^, ^52^, ^54^, ^55^, ^56^, ^58^, ^59^, ^60^, ^62^, ^63^, ^64^, ^65^, ^66^, ^69^, ^70^, ^71^, ^73^, ^74^, ^75^ Most ethnicity‐based interventions were delivered at the individual level, at all phases of the cancer continuum: diagnostic (three individual‐based interventions out of three),^71^, ^73^, ^74^ screening (18 individual‐based interventions out of 33)^22^, ^23^, ^24^, ^25^, ^26^, ^27^, ^31^, ^42^, ^48^, ^50^, ^51^, ^52^, ^54^, ^55^, ^63^, ^65^, ^66^, ^70^ and treatment (the only intervention) (Table S2).^75^ The second inequality of interest related to economic factors (22 interventions out of 57, 38.6%).^21^, ^22^, ^23^, ^27^, ^30^, ^32^, ^38^, ^40^, ^44^, ^45^, ^46^, ^47^, ^49^, ^53^, ^56^, ^57^, ^65^, ^67^, ^68^, ^70^, ^72^, ^74^ Similarly to ethnic‐related interventions, the majority of these were delivered at the individual level (19 out of 22, 86.4%) (Table S2).^21^, ^22^, ^23^, ^27^, ^30^, ^32^, ^38^, ^40^, ^44^, ^45^, ^49^, ^53^, ^57^, ^65^, ^67^, ^68^, ^70^, ^72^, ^74^ Other inequalities of interest were geographical (including remoteness and rurality)^35^, ^39^ and older age^76^ or sexual minority.^61^

Of the 56 trials, there was one non‐randomised trial,^35^ one unblinded^72^ and two single‐blinded trials,^32^, ^48^ three prospective^22^, ^65^, ^66^ and one feasibility trial.^21^ The median sample sizes of the 16 cluster randomised trials were 299 treated (interquartile range [IQR]: 162–740, representing the 25th and 75th percentiles) and 420 control individuals (IQR: 167–719), in an average of 16 and 17 clusters, respectively. The median sample sizes of the 41 individually randomised trials were 234 treated (IQR: 117–539) and 160 control individuals (IQR: 82–444).

### 3.3 Characteristics of the participants

The average recruitment period of participants was 24.1 months, ranging from an average of 22.5 months (screening) to an average of 42 months (treatment). Follow up of participants lasted for an average of 8.3 months, from an average of 5 months (interventions at diagnosis phase) up to 9 months (treatment).

Over 60% of interventions (37 out of 57) reported some contribution of the study population in designing or delivering the intervention^21^, ^22^, ^23^, ^25^, ^26^, ^28^, ^29^, ^31^, ^32^, ^33^, ^34^, ^35^, ^37^, ^39^, ^40^, ^43^, ^44^, ^47^, ^48^, ^49^, ^50^, ^51^, ^52^, ^57^, ^59^, ^60^, ^61^, ^62^, ^64^, ^65^, ^66^, ^69^, ^71^, ^72^, ^73^, ^74^; a similar proportion reported Public and Patient Involvement and Engagement (PPIE) in the design and implementation of the interventions (Table 1).^21^, ^22^, ^23^, ^24^, ^25^, ^26^, ^28^, ^29^, ^30^, ^32^, ^33^, ^34^, ^35^, ^37^, ^39^, ^40^, ^41^, ^42^, ^43^, ^45^, ^49^, ^50^, ^52^, ^54^, ^59^, ^62^, ^63^, ^64^, ^65^, ^67^, ^69^, ^71^, ^72^, ^73^, ^74^, ^75^ These proportions were highest for interventions delivered at the community level rather than at the individual level (84.6% and 76.9%, respectively, Table S2). The contributions ranged from testing the feasibility and acceptability of the intervention, co‐designing the material (print, education, and video) to participating as co‐researchers.

### 3.4 Interventions and outcomes

The interventions aimed at removing the impact of barriers that prevent some groups of the population from accessing health services. There were 53 barriers mentioned in all 57 interventions studied in this review. These included issues around knowledge(26.4%),^29^, ^37^, ^41^, ^46^, ^47^, ^52^, ^59^, ^61^, ^62^, ^64^, ^70^, ^72^, ^73^, ^74^ communication (18.9%),^26^, ^34^, ^40^, ^50^, ^51^, ^56^, ^68^, ^69^, ^73^, ^74^ financial situation (17.0%),^26^, ^32^, ^37^, ^40^, ^41^, ^46^, ^56^, ^57^, ^67^ logistics (11.3%)^37^, ^41^, ^46^, ^67^, ^73^, ^74^ and individual barriers (9.4%).^37^, ^49^, ^54^, ^61^, ^65^ Interventions also tried to improve adherence to treatment and follow‐up,^45^, ^75^ awareness,^36^ reduce distance to tests,^35^ and target beliefs and culture (Table 2).^33^, ^34^, ^43^, ^55^, ^71^

**TABLE 2:** Means/tools, barriers, outcomes, and limitations of the interventions.

	*N*	% (of each category)	% (of the 57 interventions)	% with 1‐tool intervention
**Delivery of intervention**				
Community	13	22.8		61.5
Individual	37	64.9		40.5
Both	7	12.3		42.9
Total	57	100.0		
**Means/tools**				
App	4	4.0	7.0	25.0
Decision aid	3	3.0	5.3	66.7
Home visit (s)	2	2.0	3.5	0.0
Patient navigation	11	11.1	19.3	36.4
Phone call(s)	10	10.1	17.5	10.0
Print	18	18.2	31.6	22.2
Provision of tests	14	14.1	24.6	28.6
Text message	1	1.0	1.8	100.0
Video	7	7.1	12.3	14.3
Workshop(s)	17	17.2	29.8	41.2
Other	12	12.1	21.1	8.3
Total	99	100.0		
**Barriers**				
Adherence/follow‐up	2	3.8	3.5	100.0
Awareness	1	1.9	1.8	100.0
Beliefs	3	5.7	5.3	100.0
Communication	10	18.9	17.5	30.0
Culture	2	3.8	3.5	100.0
Distance	1	1.9	1.8	0.0
Economic	9	17.0	15.8	11.1
Individual barriers	5	9.4	8.8	20.0
Knowledge	14	26.4	24.6	42.9
Logistics	6	11.3	10.5	33.3
Total	53	100.0		
**Outcomes**				
Diagnostic uptake	3	3.6	5.3	100.0
Psychological outcomes	22	26.5	38.6	50.0
Screening uptake	48	57.8	84.2	39.6
Test performances	1	1.2	1.8	100.0
Treatment initiation	2	2.4	3.5	50.0
Cost effectiveness	3	3.6	5.3	0.0
Other	4	4.8	7.0	25.0
Total	83	100.0		
**Limitations**				
Confounding	8	5.6	14.0	62.5
Individual/system barriers	6	4.2	10.5	50.0
Data quality	11	7.7	19.3	36.4
Intervention	27	18.9	47.4	40.7
Lack of generalisability	23	16.1	40.4	39.1
Outcome measurement	19	13.3	33.3	31.6
Provider	2	1.4	3.5	50.0
Lack of randomisation	1	0.7	1.8	0.0
Sample size	10	7.0	17.5	40.0
Selection bias	7	4.9	12.3	71.4
Short follow‐up	10	7.0	17.5	30.0
Study design	19	13.3	33.3	36.8
Total	143	100.0		

Varied means and tools were employed in the interventions to reduce inequalities between groups of the population. The main intervention means were the use of print material (18 interventions, 18.2% of all means reported, 31.6% of all interventions)^25^, ^30^, ^38^, ^39^, ^43^, ^44^, ^49^, ^50^, ^51^, ^54^, ^55^, ^57^, ^65^, ^67^, ^68^, ^69^, ^70^, ^71^ and workshops (17 interventions, 17.2% of all means reported, and 29.8% of all interventions).^25^, ^28^, ^29^, ^33^, ^34^, ^35^, ^37^, ^41^, ^42^, ^47^, ^56^, ^59^, ^60^, ^61^, ^62^, ^64^, ^69^ Provision of tests,^22^, ^24^, ^32^, ^35^, ^37^, ^40^, ^41^, ^46^, ^51^, ^53^, ^56^, ^58^, ^68^, ^70^ patient navigation,^41^, ^44^, ^45^, ^46^, ^56^, ^64^, ^67^, ^70^, ^73^, ^74^, ^75^ and phone calls^22^, ^30^, ^38^, ^48^, ^49^, ^50^, ^54^, ^62^, ^65^, ^70^ were reported in over 10.1% of means and in 17.5%–24.6% of interventions (Table 2).

Most intervention means were used in combinations. Of note, decision aid, workshops, and patient navigations were used on their own in 66.7%, 41.2%, and 36.4% of interventions making use of these means (Table 2). The “other means” category spanned a wide range of tools, including education of health care providers,^36^ mobile health research facility,^66^ flexible opening hours for centres,^41^ and monetary incentives.^57^ Overall, the interventions designed to address inequalities fall under the umbrella of complex (multicomponent) interventions.

Detailing the components of interventions, we can report that many targeting ethnic‐related inequalities started with educational workshops or videos, delivered to the community (20 out of 37),^23^, ^25^, ^26^, ^28^, ^29^, ^33^, ^34^, ^37^, ^41^, ^42^, ^51^, ^56^, ^59^, ^60^, ^62^, ^64^, ^66^, ^69^ which were followed, for 35.0% of them (7 out of 20) by components delivered to individuals (phone calls, mail or peer navigation)^26^, ^56^, ^62^, ^64^ or at the level of the system (low cost or free tests, changes in clinic opening times).^37^, ^41^, ^51^ Three interventions provided patient navigation services only^73^, ^74^, ^75^ and two others provided free home test kits only.^24^, ^58^

Of the 22 interventions targeting socio‐economic inequalities, most were designed to provide home or free tests for their participants (36.4%, 8 out of 22),^22^, ^32^, ^40^, ^46^, ^53^, ^56^, ^68^, ^70^ or provided education via mail, calls, or app (31.8%, 7 out of 22).^22^, ^38^, ^40^, ^49^, ^65^, ^70^, ^72^ Education workshops were only proposed in four interventions, and patient navigation in five interventions.

Considering patient navigation, free or home delivered tests as system changes, 30 interventions (52.6%) included a clear system change while the rest of them focused solely on changing patient knowledge, beliefs, or behaviours.

### 3.5 Reported outcomes and effects of the interventions

The effects of interventions were assessed on specific outcomes including screening uptake (48, 84.2% of interventions)^21^, ^22^, ^23^, ^24^, ^25^, ^26^, ^27^, ^29^, ^30^, ^31^, ^32^, ^33^, ^34^, ^35^, ^36^, ^37^, ^38^, ^39^, ^40^, ^41^, ^42^, ^43^, ^44^, ^45^, ^46^, ^47^, ^49^, ^50^, ^51^, ^52^, ^54^, ^55^, ^56^, ^57^, ^58^, ^59^, ^60^, ^61^, ^62^, ^63^, ^64^, ^65^, ^66^, ^67^, ^68^, ^69^, ^70^ and psychological outcomes (e.g., acceptability of the intervention, behaviour change, and symptom recognition; 22, 38.6% of interventions).^23^, ^26^, ^27^, ^28^, ^29^, ^31^, ^33^, ^35^, ^37^, ^39^, ^48^, ^55^, ^56^, ^59^, ^60^, ^61^, ^62^, ^63^, ^64^, ^66^, ^71^, ^72^ Other outcomes, used in less than five interventions, included diagnostic uptake^24^, ^73^, ^74^ and treatment initiation,^75^, ^76^ test performance,^53^ and cost effectiveness (Table 2).^25^, ^26^, ^72^

Overall, 68.4% of interventions reported evidence of a positive benefit on their primary outcome,^22^, ^24^, ^25^, ^26^, ^28^, ^29^, ^30^, ^31^, ^34^, ^35^, ^36^, ^38^, ^40^, ^41^, ^45^, ^46^, ^47^, ^49^, ^50^, ^51^, ^52^, ^53^, ^57^, ^58^, ^59^, ^60^, ^61^, ^62^, ^63^, ^64^, ^65^, ^66^, ^67^, ^68^, ^69^, ^70^, ^73^, ^75^, ^76^ with a larger proportion for interventions delivered at the community level (76.9%) than interventions delivered at the individual level (64.9%). Only 25.0% (1 out of 4) of interventions had positive benefits on their primary outcome at the diagnosis phase while 70.6% of the screening interventions and 100.0% (2 out of 2) of the treatment interventions reported a positive benefit on primary outcomes—these differences mostly reflect the different number of interventions in each phase. Almost half of the interventions did not measure or report any impact on secondary outcomes. Of those that did, 18 out of 31 (58.1%) reported evidence of a benefit,^21^, ^31^, ^34^, ^36^, ^38^, ^45^, ^47^, ^48^, ^52^, ^57^, ^59^, ^60^, ^61^, ^62^, ^64^, ^70^, ^72^, ^76^ with similarly lower proportions of interventions delivered at the individual level compared to the community level (Table S2).

### 3.6 Challenges and limitations of the interventions

There were many types of limitations reported on the tested interventions, ranging from the intervention itself (e.g., contamination across study groups, setting; 27 interventions, 47.4%),^21^, ^22^, ^25^, ^27^, ^28^, ^29^, ^31^, ^35^, ^38^, ^39^, ^40^, ^43^, ^51^, ^53^, ^56^, ^59^, ^63^, ^64^, ^65^, ^66^, ^67^, ^70^, ^72^, ^74^, ^75^, ^76^ a lack of generalisability (23 interventions, 40.4%),^22^, ^24^, ^26^, ^28^, ^29^, ^30^, ^34^, ^39^, ^40^, ^42^, ^43^, ^49^, ^52^, ^57^, ^58^, ^60^, ^62^, ^64^, ^66^, ^67^, ^69^, ^70^, ^72^ study design (e.g., no randomisation, lack of blinding; 19 interventions, 33.3%),^24^, ^30^, ^31^, ^35^, ^37^, ^39^, ^40^, ^41^, ^44^, ^46^, ^48^, ^49^, ^52^, ^53^, ^56^, ^58^, ^63^, ^69^, ^76^ outcome measurement (e.g., recall bias for self‐reported measures, internal consistency; 19 interventions, 33.3%),^25^, ^29^, ^37^, ^40^, ^42^, ^48^, ^50^, ^52^, ^54^, ^58^, ^61^, ^62^, ^63^, ^65^, ^66^, ^67^, ^69^, ^72^, ^75^ data quality (11 interventions, 19.3%),^22^, ^26^, ^44^, ^45^, ^49^, ^50^, ^60^, ^65^, ^74^, ^75^, ^76^ sample size (10 interventions, 17.5%),^21^, ^29^, ^35^, ^36^, ^38^, ^51^, ^65^, ^67^, ^68^ short follow‐up (10 interventions, 17.5%)^25^, ^29^, ^35^, ^36^, ^41^, ^56^, ^59^, ^60^, ^66^, ^76^ and selection bias (7 interventions, 12.3%) (Table 2).^51^, ^52^, ^55^, ^64^, ^71^, ^73^, ^74^

## 4 DISCUSSION

The insights from this scoping review of the literature on controlled evaluation of interventions specifically aimed at reducing inequalities along the cancer care pathway showed a clear lack of diversity in many aspects: 89.5% of the interventions focused on access to screening, 98.2% targeted colorectal, breast, and cervical cancers, 64.9% concentrated on ethnic inequalities, and 84.2% were based in the USA. In addition, a large number of interventions aimed to change individual knowledge, beliefs, and behaviour rather than targeted system‐level factors where many inequalities stem from.

On the whole, it appears that ethnic‐related inequalities are mostly addressed with educational workshops and peer navigation, while socio‐economic inequalities are addressed by easier access to tests (remove costs/home‐test kits) or by offering educational material (leaflets, calls, and apps). This may suggest that ethnic inequalities seem envisaged through the lens of a knowledge gap rather than considering the potential role of institutional barriers to access.

Though inequalities in the cancer care pathway do start from the point of seeking help in primary healthcare settings (e.g., symptom recognition, risk‐taking behaviours, and increased used of emergency care),^3^, ^11^ or taking up screening,^9^, ^10^ it is well documented that they do persist and accumulate throughout the diagnostic process, treatment, and post‐treatment phases.^1^, ^2^, ^14^ These appear neglected in the literature on interventions, such that we found two articles only that focused on the treatment phase.^75^, ^76^ The focus on addressing inequalities in ‘entry’ to the cancer pathway likely reflects a concern with intervening where the impact would be greatest. The scarcity of interventions during the cancer treatment phase could point to a relatively neglected area of experimental research for which the reasons may be complex. However, the success of interventions that aim to improve earlier diagnosis of cancer for all patients, and particularly for those from backgrounds known to miss out, needs the additional support of (i) appropriate downstream medical services (e.g., pathology), (ii) free and accessible diagnostic and treatment services for those who cannot afford to continue receiving medical care, and (iii) sustainable and cost‐effective approaches. These downstream aspects were not described in the literature we reviewed.

This review was designed to study experimental or ongoing interventions in the published literature. We are aware that many actions, interventions, and changes aimed at reducing inequalities in access to cancer care and in outcomes may be tested and evaluated as part of local policy priorities and directives. It would be very informative to complement the learnings of this review with a study of the grey literature spanning a smaller geographical area; this would bring more diversity in the design and operationalisation of interventions. Other reviews related to inequalities in cancer have been published in the past few years but focused on specific populations and interventions, which do not encompass the breadth of research in this area. Nevertheless, our findings are consistent with published findings, despite their different methodologies, search strategy, and focus.^77^, ^78^, ^79^, ^80^

Inequalities in access to adequate and optimal care exist in countries other than the USA, even where universal health insurance is available.^13^ While these inequalities can only be recognised in systems with reliable data, developing adequate cancer policies—adapted to national and local contexts—are essential though they need to embed an equity lens specifically addressing the generation or amplification of inequalities.^19^

All interventions studied in this review correspond to ‘vertical’ interventions in that (i) they all target a specific, well‐identified inequality, (ii) individuals receiving the interventions are well specified based on socio‐demographic characteristics such as their country of origin, mother tongue, place of residence, age, sexual orientation, (iii) the interventions are very specific to the individuals targeted (development of material in a specific language, lay navigators from the community). The outcomes of interest were measured in the short‐term following the intervention, with little impact on the larger community around individual participants. In contrast, horizontal approaches, tackling the overall inequalities in distribution, access and use of cancer services, would provide more long term and sustainable approaches to addressing social determinants of health.

Many studies in this review did not appear to follow the standards for experimental research. In particular, some articles reported that their interventions suffered from various limitations due to, for example, the design of their trial, their sample size, confounding and selection biases, and lack of systematic and unbiased outcome measurements. Some studies may have been underpowered due to the lack of appropriate sample size calculation, and it became clear during the data extraction process that CONSORT guidelines for the reporting of randomised studies were rarely used. Research on the reduction of inequalities in cancer care and outcomes would benefit from improved design, rigour, and appropriate evaluation. The predominance of studies from the USA in this review may reflect a lack of relevant studies elsewhere or the consequence of our search strategy and database selection. While we restricted our review to English‐language articles, it is possible that relevant non‐English studies meeting our inclusion criteria were missed. In addition, our reliance on two databases, including Medline, which has a strong focus on USA publications, may have contributed to this bias. Furthermore, the focus on OECD countries in our search strategy may have inadvertently excluded studies where country information was not explicitly stated in the abstract. Another limitation is the influence of publication bias. It is possible that interventions targeting certain aspects of the cancer care continuum or other types of inequalities, possibly more subtle or with less straightforward mechanisms, have been trialled but were not published due to lack of results or unfavourable outcomes.

In this review, we purposefully focused on direct inequalities along the cancer care pathway, as these are large and persisting. Nonetheless, we aim to acknowledge here other ongoing, key and varied research, highly correlated to our primary focus: (i) diversity and representation of patients selected for clinical trials,^81^, ^82^, ^83^, ^84^, ^85^ essential to bring knowledge on best treatment and care for different populations; (ii) inequalities at the survivorship phase, such as in access to financial products for cancer survivors,^86^ and (iii) closing the gap in international disparities in cancer care.^87^

The interventions described in this review mostly target individuals with expectations that they will be better informed (‘educated’) or adhere and engage with healthcare in new ways. Very few interventions tackled factors referring to how healthcare is delivered, which may reinforce inequalities. Exceptions included examples of mobile screening trucks in remote areas or education of healthcare practitioners. While individuals should make informed decisions and be aware of cancer‐related risk factors and opportunities for screening and early diagnosis, it is important to recognise and alleviate the burden that rests on individuals, prior to and instead of adding to it. While education is a critical component of health interventions, evidence from other contexts, such as medication adherence^88^ suggests that educational interventions alone are often insufficient to drive meaningful behaviour change. This raises important questions about the long term effectiveness of education‐based approaches in addressing cancer inequalities. Future interventions may need to incorporate an intersectional approach that accounts for concurrent and interacting influences on how inequalities arise along the cancer care pathway and how certain groups of people are disadvantaged.

## AUTHOR CONTRIBUTIONS

**Wende C. Safari:** Conceptualization; methodology; validation; writing – review and editing; writing – original draft; formal analysis. **Katja Gravenhorst:** Conceptualization; methodology; validation; formal analysis; writing – review and editing. **Clemence Leyrat:** Conceptualization; validation; methodology; formal analysis; writing – review and editing. **Koki Shimizu:** Conceptualization; methodology; validation; formal analysis; writing – review and editing. **Matthew J. Smith:** Methodology; validation; writing – review and editing; formal analysis. **Ajay Aggarwal:** Conceptualization; writing – review and editing. **Camille Maringe:** Conceptualization; methodology; validation; formal analysis; supervision; writing – original draft; writing – review and editing.

## FUNDING INFORMATION

Cancer Research UK C7923/A29018.

## CONFLICT OF INTEREST STATEMENT

The authors declare no conflicts of interest.

## Supporting information


**APPENDIX S1:** Search terms.
**APPENDIX S2:** Preferred reporting items for systematic reviews and meta‐analyses extension for scoping reviews (PRISMA‐ScR) checklist.
**APPENDIX S3:** Protocol—v1.2.
**APPENDIX S4:** Extraction list.

**TABLE S1:** Overview of the 56 studies.
**TABLE S2:** General description of the 57 interventions studied by cancer phase and intervention type.

## Data Availability

The data that support the findings of this study are available from the corresponding author upon reasonable request.
