# Supplementary material for: Characteristics of interventions aimed at reducing inequalities along the cancer continuum: A scoping review
Source: Int J Cancer. 2025 May 26;157(6):1043–54. doi: 10.1002/ijc.35478 (PMC12280855; doi:10.1002/ijc.35478)
Supplement: Supplementary file 1 — APPENDIX S1: Search terms. APPENDIX S2: Preferred reporting items for systematic reviews and meta‐analyses extension for scoping reviews (PRISMA‐ScR) checklist. APPENDIX S3: Protocol—v1.2. APPENDIX S4: Extraction list. [file IJC-157-1043-s001.pdf]

Supplementary materials

Characteristics of interventions aimed at reducing inequalities along the cancer continuum: a scoping review

Wende Safari, Katja Gravenhorst, Clemence Leyrat, Koki Shimizu, Matthew Smith, Ajay Aggarwal, Camille Maringe

Content:

Appendix 1. Search terms

Appendix 2. Preferred Reporting Items for Systematic reviews and Meta-Analyses extension for Scoping Reviews (PRISMA-ScR) Checklist

Appendix 3. Protocol – v1.2

Appendix 4. Extraction list

Appendix Table 1. Overview of the 56 studies (uploads as a separate excel file)

Appendix Table 2. General description of the 57 interventions studied by cancer phase and intervention type (uploads as a separate excel file)

Appendix 1. Search terms

Intervention

1. Intervent*[tiab] OR approach*[tiab] OR strateg*[tiab] OR plan*[tiab] OR pilot*[tiab] OR program*[tiab] OR initiative*[tiab]
2. "Health Promotion"[Mesh] OR "Early medical intervention"[Mesh]
3. 1 OR 2

Inequalities

1. Inequal*[tiab] OR inequit*[tiab] OR disparit*[tiab] OR depriv*[tiab] OR equit*[tiab] OR minorit*[tiab] OR disadvantage*[tiab] OR vulnerable[tiab] OR variation*[tiab] OR underserved[tiab] OR poor[tiab]
2. "Health Status Disparities"[Mesh] OR "Healthcare Disparities"[Mesh] OR "Health Inequities"[Mesh] OR "Vulnerable Populations"[Mesh] OR "Poverty Areas"[Mesh] OR "Minority Groups"[Mesh]
3. 4 OR 5

Solid tumours

1. Tumo?r[tiab] OR malignanc*[tiab] OR oncol*[tiab] OR neoplasm*[tiab] OR cancer*[tiab] OR melanoma*[tiab] OR sarcoma*[tiab] OR carcinoma*[tiab]
2. Neoplasms[Mesh] OR "Palliative Care"[Mesh]
3. 7 OR 8
4. 3 AND 6 AND 9

Embase:

1. 'adaptive clinical trial (topic)'/de OR 'adaptive clinical trial'/de OR 'clinical trial (topic)'/de OR 'clinical trial'/de OR 'controlled clinical trial (topic)'/de OR 'controlled clinical trial'/de OR 'double blind procedure'/de OR 'early termination of clinical trial'/de OR 'equivalence trial (topic)'/de OR 'equivalence trial'/de OR 'intention to treat analysis'/de OR 'multicenter study (topic)'/de OR 'multicenter study'/de OR 'non-inferiority trial'/de OR 'phase 1 clinical trial (topic)'/de OR 'phase 1 clinical trial'/de OR 'phase 2 clinical trial (topic)'/de OR 'phase 2 clinical trial'/de OR 'phase 3 clinical trial (topic)'/de OR 'phase 3 clinical trial'/de OR 'phase 4 clinical trial (topic)'/de OR 'phase 4 clinical trial'/de OR 'pragmatic trial'/de OR 'randomized controlled trial (topic)'/de OR 'randomized controlled trial'/de OR 'superiority trial'/de OR 'multicenter study':ti,ab,kw OR 'phase I':ti,ab,kw OR 'phase II':ti,ab,kw OR 'phase III':ti,ab,kw OR 'phase IV':ti,ab,kw OR 'phase 1':ti,ab,kw OR 'phase 2':ti,ab,kw OR 'phase 3':ti,ab,kw OR 'phase 4':ti,ab,kw OR ((randomised OR randomized) NEAR/7 trial*) OR (controlled NEAR/3 trial*) OR (clinical NEAR/2 trial*) OR ((single:ti,ab,kw OR doubl*:ti,ab,kw OR tripl*:ti,ab,kw OR treb*:ti,ab,kw) and (blind*:ti,ab,kw OR mask*:ti,ab,kw)) OR '4 arm':ti,ab,kw OR 'four arm':ti,ab,kw

Medline

2. ((("randomized controlled trial"[pt] OR "controlled clinical trial"[pt] OR "randomized"[tiab] OR "placebo"[tiab]) OR ("clinical trials as topic" [mesh:noexp]) OR (randomly [tiab] OR trial [ti])) NOT (animals [mh] NOT humans [mh]))

3. ("adaptive clinical trial" or "clinical trial" or "clinical trial, phase i" or "clinical trial, phase ii" or "clinical trial, phase iii" or "clinical trial, phase iv" or "controlled clinical trial" or "equivalence trial" or "multicenter study" or "pragmatic clinical trial" or "randomized controlled trial").pt. or double-blind method/ or "adaptive clinical trials as topic"/ or "clinical trials as topic"/ or "clinical trials, phase i as topic"/ or "clinical trials, phase ii as topic"/ or "clinical trials, phase iii as topic"/ or "clinical trials, phase iv as topic"/ or "controlled clinical trials as topic"/ or "equivalence trials as topic"/ or "intention to treat analysis"/ or "non-randomized controlled trials as topic"/ or "pragmatic clinical trials as topic"/ or "randomized controlled trials as topic"/ or "multicenter studies as topic"/ or ((phase adj1 ("I" or "II" or "III" or "IV" or "1" or "2" or "3" or "4"))).ti,ab,kf. or ((randomi?ed adj7 trial*) or (controlled adj3 trial*) or ((clinical or pragmatic) adj2 trial*) or (research adj (studies or study)) or ((single or doubl* or tripl* or treb*) adj4 (blind* or mask*))).ti,ab,kf. or (("4" or four) adj arm).ti,ab,kf.

OECD countries

(exp canada/ or exp mexico/ or exp united states/ or chile/ or costa rica/ or colombia/ or israel/ or "Turkey (republic)"/ or exp japan/ or south korea/ or austria/ or exp belgium/ or benelux/ or exp baltic states/ or estonia/ or latvia/ or lithuania/ or czech republic/ or hungary/ or poland/ or slovakia/ or slovenia/ or exp france/ or exp germany/ or exp united kingdom/ or exp "great britain" or england/ or northern ireland/ or exp scotland/ or wales/ or greece/ or exp ireland/ or exp italy/ or luxembourg/ or netherlands/ or portugal/ or exp Scandinavia/ or exp denmark/ or exp finland/ or iceland/ or exp norway/ or sweden/ or exp spain/ or switzerland/ or exp Australia and New Zealand/ or exp australia/ or exp new zealand/ OR (Australia or Austria or Belgium or Canada or Chile or Colombia or "Costa Rica" or "Czech Republic" or Denmark or Estonia or Finland or France or Germany or Greece or Hungary or Iceland or Israel or Italy or Japan or "Republic of Korea" or "South Korea" or Latvia or Lithuania or Luxembourg or Mexico or Netherlands or New Zealand or Norway or Poland or Portugal or "Slovak Republic" or Slovenia or Spain or Sweden or Switzerland or Turkey or "United Kingdom" or England or Ireland or Scotland or Wales or "United States").mp

Appendix 2. Preferred Reporting Items for Systematic reviews and Meta-Analyses extension for Scoping Reviews (PRISMA-ScR) Checklist

SECTION	ITEM	PRISMA-ScR CHECKLIST ITEM	REPORTED ON PAGE #
**TITLE**			
Title	1	Identify the report as a scoping review.	Yes - title
**ABSTRACT**			
Structured summary	2	Provide a structured summary that includes (as applicable): background, objectives, eligibility criteria, sources of evidence, charting methods, results, and conclusions that relate to the review questions and objectives.	Inappropriate for the journal that requires a non-structure abstract
**INTRODUCTION**			
Rationale	3	Describe the rationale for the review in the context of what is already known. Explain why the review questions/objectives lend themselves to a scoping review approach.	Yes – end of introduction section + rationale in methods
Objectives	4	Provide an explicit statement of the questions and objectives being addressed with reference to their key elements (e.g., population or participants, concepts, and context) or other relevant key elements used to conceptualize the review questions and/or objectives.	Yes – methods section
**METHODS**			
Protocol and registration	5	Indicate whether a review protocol exists; state if and where it can be accessed (e.g., a Web address); and if available, provide registration information, including the registration number.	Review protocol attached as an appendix as was not relevant to publish online on PROSPERO that online registers systematic review
Eligibility criteria	6	Specify characteristics of the sources of evidence used as eligibility criteria (e.g., years considered, language, and publication status), and provide a rationale.	See ‘articles selection’ paragraph
Information sources*	7	Describe all information sources in the search (e.g., databases with dates of coverage and contact with authors to identify additional sources), as well as the date the most recent search was executed.	See ‘articles selection’ paragraph
Search	8	Present the full electronic search strategy for at least 1 database, including any limits used, such that it could be repeated.	See Appendix 1
Selection of sources of evidence†	9	State the process for selecting sources of evidence (i.e., screening and eligibility) included in the scoping review.	See ‘articles selection’ paragraph
Data charting process‡	10	Describe the methods of charting data from the included sources of evidence (e.g., calibrated forms or forms that have been tested by the team before their use, and whether data charting was done independently or in duplicate) and any processes for obtaining and confirming data from investigators.	See ‘data extraction’ paragraph

SECTION	ITEM	PRISMA-ScR CHECKLIST ITEM	REPORTED ON PAGE #
Data items	11	List and define all variables for which data were sought and any assumptions and simplifications made.	See 'data extraction' paragraph
Critical appraisal of individual sources of evidence§	12	If done, provide a rationale for conducting a critical appraisal of included sources of evidence; describe the methods used and how this information was used in any data synthesis (if appropriate).	NA
Synthesis of results	13	Describe the methods of handling and summarizing the data that were charted.	See 'data extraction' paragraph
**RESULTS**			
Selection of sources of evidence	14	Give numbers of sources of evidence screened, assessed for eligibility, and included in the review, with reasons for exclusions at each stage, ideally using a flow diagram.	See 'search and selection of articles' paragraph
Characteristics of sources of evidence	15	For each source of evidence, present characteristics for which data were charted and provide the citations.	See 'characteristics of the studies' paragraph
Critical appraisal within sources of evidence	16	If done, present data on critical appraisal of included sources of evidence (see item 12).	NA
Results of individual sources of evidence	17	For each included source of evidence, present the relevant data that were charted that relate to the review questions and objectives.	Results section
Synthesis of results	18	Summarize and/or present the charting results as they relate to the review questions and objectives.	See Tables 1 and 2
**DISCUSSION**			
Summary of evidence	19	Summarize the main results (including an overview of concepts, themes, and types of evidence available), link to the review questions and objectives, and consider the relevance to key groups.	Start of the discussion
Limitations	20	Discuss the limitations of the scoping review process.	Done in the middle of the discussion
Conclusions	21	Provide a general interpretation of the results with respect to the review questions and objectives, as well as potential implications and/or next steps.	Discussion
**FUNDING**			
Funding	22	Describe sources of funding for the included sources of evidence, as well as sources of funding for the scoping review. Describe the role of the funders of the scoping review.	Done in the funding section

JBI = Joanna Briggs Institute; PRISMA-ScR = Preferred Reporting Items for Systematic reviews and Meta-Analyses extension for Scoping Reviews.

* Where *sources of evidence* (see second footnote) are compiled from, such as bibliographic databases, social media platforms, and Web sites.

† A more inclusive/heterogeneous term used to account for the different types of evidence or data sources (e.g., quantitative and/or qualitative research, expert opinion, and policy documents) that may be eligible in a scoping review as opposed to only studies. This is not to be confused with *information sources* (see first footnote).

‡ The frameworks by Arksey and O'Malley (6) and Levac and colleagues (7) and the JBI guidance (4, 5) refer to the process of data extraction in a scoping review as data charting.

§ The process of systematically examining research evidence to assess its validity, results, and relevance before using it to inform a decision. This term is used for items 12 and 19 instead of "risk of bias" (which is more applicable to systematic reviews of interventions) to include and acknowledge the various sources of evidence

that may be used in a scoping review (e.g., quantitative and/or qualitative research, expert opinion, and policy document).

From: Tricco AC, Lillie E, Zarin W, O'Brien KK, Colquhoun H, Levac D, et al. PRISMA Extension for Scoping Reviews (PRISMAScR): Checklist and Explanation. Ann Intern Med. 2018;169:467–473. doi: 10.7326/M18-0850.

Appendix 3. Protocol – v1.2
Scoping review of the literature on interventions aiming to reduce cancer inequalities

1. *** Review title**

Characteristics of Interventions aimed at reducing inequalities along the cancer continuum

2. **Original language title.**

English

3. *** Anticipated or actual start date.**

11th March 2024

4. *** Anticipated completion date.**

End of April 2024

5. *** Stage of review at time of this submission.**

Protocol writing

11. *** Review team members and their organisational affiliations.**

Katja Gravenhorst; Clemence Leyrat; Wende Safari; Koki Shimizu; Matthew Smith; Camille Maringe

LSHTM, HSRP

15. *** Review question.**

What are the experimentally assessed health service interventions designed to reduce cancer inequalities?

By *inequalities*, we consider unequal access to cancer services, in relation to someone's socioeconomic background, ethnicity, sex, age, comorbidities, etc. We include screening, diagnosis (primary and secondary care), treatment, post-treatment follow-up and palliative care in what we consider cancer services along the cancer journey. We exclude inequalities in secondary effects of cancer diagnosis or treatment, e.g. financial catastrophe, mental health, infertility. We do not consider regional inequalities.

By *interventions*, we refer to any changes in service delivery of screening, diagnosis, treatment, post-treatment follow up and palliative care that involved a concrete practical change in e.g. behaviour, job description, delivery or offer of a service, etc.

16. *** Searches.**

See appendix

Search strategies and libraries

We will search the PubMed database, as well as Embase, Cochrane and medrxiv.org. We will search through the references of selected publications (snowballing).

If any of the references is not open access, we will contact the corresponding author.

18. *** Condition or domain being studied.**

We study the entire cancer care pathway, focusing on the target populations for cancer screening (cervical, breast, colorectal, lung and prostate), and on adult patients with cancer at the time of

diagnosis, treatment and follow-up. We only include solid cancers and exclude blood cancers (leukemia, lymphoma, myeloma).

19. * Participants/population.

Different phases along the cancer continuum lead to different population of interest:

- Screening and early diagnosis phases: Participants are the target population from the general population
- Early diagnosis, treatment, follow-up and palliative care phases: Participants are adult cancer patients with characteristic(s) that might make them less likely to benefit from or use cancer services, resulting in suboptimal or less desirable outcomes.

20. * Intervention(s), exposure(s)

We are interested in describing and summarizing key features of all types of (i) practical interventions, (ii) assessed through routine changes or in an experimental context, (iii) aimed at reducing inequalities in access to cancer care or in cancer outcomes, (iv) in any high-income setting.

We exclude (i) policy-based interventions, defined in policy documents (e.g. cancer plans, at national or regional levels), (ii) interventions based on inequalities between countries, (iii) interventions without inequalities stated as primary outcome, or if (iv) interventions with no empirical evaluation.

21. * Comparator(s)/control.

No intervention, active controls or no comparator (e.g. before/after design)

22. * Types of study to be included.

We will consider both observational and experimental study designs with a qualitative or quantitative evaluation of the intervention. We will not consider opinion pieces, letters or editorials.

23. Context.

24. * Main outcome(s).

The review aims to provide an exhaustive overview of the interventional research on cancer inequalities: type, extent and quantity of research available.

We will assess the quality of the reporting and evaluation of the interventions.

We will collect and describe the design, implementation, setting, target population, target inequalities, how well the intervention was received, whether it was rolled out further, their limitations, outcome etc. to provide an overview of key lessons learnt, and how these may apply to the British context.

25. * Additional outcome(s).

We will collect additional information on the intervention under study (based on the TIDieR checklist): Name, description, rationale, physical or informational materials used in the intervention, description of each of the procedures, activities, and/or processes used in the intervention, expertise needed, any specific training given, modes of delivery (e.g. face to face, internet or telephone), location(s) where

the intervention occurred (specific infrastructure), adherence or fidelity (if assessed), was the intervention delivered as planned?

We will study the above-mentioned outcomes by cancer type, type of inequality, type of intervention (patient, hospital, community, local areas, etc.), by phase along the cancer pathway (screening, early diagnosis, treatment and follow-up), by broad geographical area (continent?).

26. * Data extraction (selection and coding).

**Intervention**	**Patients/population**	**Outcomes/assessment of intervention**	**Lessons learnt?**
ID number, Authors, title, year of publication	Type of inequality	Primary/secondary outcome<br>Principal results (positive, negative, NS, for each outcome)	Main limitations highlighted
Aim of study<br>Study design<br>Protocol/registration<br>TIDieR items	Socio-demo characteristics of target population<br>Eligibility criteria for population	Result of evaluation (roll out, …)	Main conclusions and recommendations
Period of time when intervention was active<br>Sample size<br><br>Cancer phase	Country/region<br>Place where study population was recruited (community, health centre, hospital…)		
Type of intervention (medical/non-medical; Individual-directed interventions; Access-enhancing interventions; Peer navigator-related interventions; Community education; Mass media; Other)	Cancer type		
	Phase along cancer continuum		

Two independent researchers will be screening each article, and two independent researchers will perform data extraction. Adjudication will be done by a third independent researcher.

We will test the extraction sheet on 5 selected articles, chosen for their differing focus: different phases along the cancer continuum, different inequalities, different types of intervention.

27. * Risk of bias (quality) assessment.

We will use the PRISMA guidelines for reporting of our literature review results.

We will focus on interventions that state inequalities as a primary outcome. We will use the SQUIRE framework for the evaluation of the reporting of new knowledge on healthcare improvement. We will use the CONSORT for reporting of RCTs, and STROBE for reporting of observational studies.

28. * Strategy for data synthesis.

Data will be synthesised in a narrative format with summary statistics of the characteristics of the interventions, populations and settings of the reviewed articles.

29. * Analysis of subgroups or subsets.

Results will be presented by phase along the cancer care journey.

30. * Type and method of review.

Type of review: Scoping review

Health area of the review: Cancer

Main focus: Interventions targeting inequalities, stated as primary outcome

31. Language.

English

32. * Country.

England

Appendix 4. Extraction list

Article

Article ID
Authors
Title
Year of publication
Journal
DOI

Exclusions

Exclusion [Y or blank]
Reason for exclusion
Literature review [Y or blank]

Study

Aim of study (as stated)
Study design as described (e.g. RCT, quasi-experimental)
Design type as described (free text: step-wedge, cross-over, cluster…)
Protocol available [Y/N]
    Protocol reg. number, when available

Setting

Dates of recruitment of participants
Duration of follow-up
Multi-centric [Y/N] (Y if there was more than one study site (mobile unit would not be multicentric as it represents one delivery unit only)
International [Y/N]
Geographic location (country)
Geographic location (region/city) - list if <10
Setting of delivery of intervention (e.g. hospital, primary care, community, …)
Sample size at recruitment: intervention arm (n or NA)
Sample size at recruitment: control arm (n or NA)

Sample characteristics

Number of cancer sites studied
Cancer sites (list all)
Age-group
Sex
Socio-economic level [anything that gives indication of SES: education, insurance status, index of deprivation…]
Specific comorbidity(ies)
Specific ethnic group(s)
Geographical characteristics [e.g., rural, remote, urban, peripheral, …]
Any other characteristics
Place where study participants were recruited (e.g. community, hospital, health centre, GP practice, no active recruitment)

Which characteristic/inequality is tackled by the intervention? [e.g. age, ethnicity, SES, geography,…]

Intervention

Is there any information on whether the study population contributed to any part of the project? [Y/N]

How was the study population involved? (e.g., RQ, design, recruitment, dissemination of results, other?)

Phase of cancer continuum [pre-diagnosis (incl. awareness), screening, diagnosis, treatment, follow-up, palliative care]

Level of the intervention [individual, community, both]

Type of intervention [individual directed; access-enhancing; peer-navigator related; community education; mass media; … other]

Description of the intervention (text from article)

Comparator [no active comparator (usual care), active control]

Description of usual care

Primary outcome

Secondary outcome(s)

Results and discussion

Principal results (primary outcome): Evidence of a benefit [Y/N]

Principal results (secondary outcome(s)): Evidence of a benefit [Y/N]

Conclusion(s) from authors - text that reports main results on outcomes of interest

Limitations as highlighted by authors

Recommendations from authors

Funding [private, public, both]

Linked studies/plans/results

Any work/public engagement/study/publications prior to this study? [Y/N]

What work/public engagement/study/publications prior to this study?

Any follow up study/plans? [Y/N]

What follow up study/plans?

Quality

Followed CONSORT [Y, N, unclear]

Overall quality [Good, Medium, Bad]

Comment on quality
